# Modification of optical and electrical properties of zinc oxide-coated porous silicon nanostructures induced by swift heavy ion

**DOI:** 10.1186/1556-276X-7-366

**Published:** 2012-07-02

**Authors:** Yogesh Kumar, Manuel Herrera-Zaldivar, Sion Federico Olive-Méndez, Fouran Singh, Xavier Mathew, Vivechana Agarwal

**Affiliations:** 1 Centro de Investigacion en Ingenieria y Ciencias Aplicadas, UAEM, Av. Univ. 1001, Col. Chamilpa, Cuernavaca, Morelos, 62209, México; 2 Materials Science Group, Centro de Nanociencia y Nanotecnología, UNAM, Ensenada Apdo, Postal 14, Ensenada, Baja California, 22800, México; 3 Inter University Accelerator Centre, Aruna Asaf Ali Marg, New Delhi, 110067, India; 4 Centro de Investigacion in Materiales Avanzados, Ave. Miguel de Cervantes 120, Complejo Industrial Chihuahua, Chihuahua, 31109, México; 5 Centro de Investigacion Energia, UNAM, Privada Xochicalco S/N, Temixco, Morelos, 62580, México

**Keywords:** Porous silicon, Zinc aluminum oxide, Swift heavy ions, Photoluminescence, Cathodoluminescence.

## Abstract

Morphological and optical characteristics of radio frequency-sputtered zinc aluminum oxide over porous silicon (PS) substrates were studied before and after irradiating composite films with 130 MeV of nickel ions at different fluences varying from 1 × 10^12^ to 3 × 10^13^ ions/cm^2^. The effect of irradiation on the composite structure was investigated by scanning electron microscopy, X-ray diffraction (XRD), photoluminescence (PL), and cathodoluminescence spectroscopy. Current–voltage characteristics of ZnO-PS heterojunctions were also measured. As compared to the granular crystallites of zinc oxide layer, Al-doped zinc oxide (ZnO) layer showed a flaky structure. The PL spectrum of the pristine composite structure consists of the emission from the ZnO layer as well as the near-infrared emission from the PS substrate. Due to an increase in the number of deep-level defects, possibly oxygen vacancies after swift ion irradiation, PS-Al-doped ZnO nanocomposites formed with high-porosity PS are shown to demonstrate a broadening in the PL emission band, leading to the white light emission. The broadening effect is found to increase with an increase in the ion fluence and porosity. XRD study revealed the relative resistance of the film against the irradiation, i.e., the irradiation of the structure failed to completely amorphize the structure, suggesting its possible application in optoelectronics and sensing applications under harsh radiation conditions.

## Background

Nowadays, efforts are being made to look for suitable types of nanocomposites for optoelectronic applications. Semiconductor nanocrystallites have been considered as the emission source for the next-generation light-emitting diodes due to their electro-optical properties and tunable size [1,2]. Zinc oxide (ZnO) with trivalent elements such as aluminum (Al) is a unique n-type semiconductor and transparent material with a direct bandgap of 3.37 eV, along with a large exciton binding energy of 60 meV [3]. Al-doped ZnO (AZO) is considered as an important material for its application as a transparent electrode in flat panel displays [4] due to its high conductivity and good transparency. Till now, AZO films with resistivity lower than 1 to 5 × 10^−4^ Ω cm [5-7] and transmittance more than 85 % have been attained. On the other hand, porous silicon (PS) has been investigated due to its room-temperature luminescence, and efforts have been focused to obtain an efficient electroluminescent (EL) device [8] based on PS for its possible integration with the present microelectronic industry. Along with various semiconducting and piezoelectric properties suitable for various applications in the optoelectronic industry, the EL efficiency could be increased strongly by filling the pores of PS with AZO. Due to the open structure and large surface area, together with the unique optical properties, PS is a good candidate for a template. It is known that the emission energy of PS increases with a decrease in silicon nanocrystallite size covering the entire visible spectrum from red to blue [9]. It has been reported that the blue luminescence band with a relatively fast decay time is observed in the oxidized PS samples. On the other hand, it is easy to get the red emission from the PS, and if it could be added with any other semiconductor with emission in the blue green region, it could be useful to obtain the white light through a simple route for possible applications like the display technology [4,10]. Apart from that, our group recently demonstrated the importance of PS-ZnO composites for sensing application through a control over the spacial distribution of zinc oxide and its transport properties on the porosity of the PS substrate [11].

In the last few years, a considerable amount of progress has been done to enhance the optical properties and other physical characteristics of the ZnO film with different techniques. Among them, energetic ion beams have been employed to modify the electrical, optical, and structural properties of different materials [12-15]. Matsunami et al. [16] studied the effect of irradiation on the electrical, structural, and optical properties of indium-doped ZnO films and found an enhancement in the electrical conductivity. Sugai et al. [17] reported a two-order increase in the conductivity of AZO films after irradiation with Ni and Xe ions. Recently, Singh et al. [18] have reported an increase in the ethanol sensing response of irradiated SnO_2_ films with ZnO demonstrating a strong resistance to damage caused by ion irradiation. Another work on ZnO-PS nanocomposites [19], where ZnO deposited onto the microporous silicon with sol–gel technique, showed the suppression of X-ray diffraction (XRD) peaks after irradiating it with heavy ions (Au). Hence, the effect of high-energy light ions (such as nickel) could be interesting in studying the stability of the ZnO structure along with its optical properties. In this work, we have investigated the ion irradiation effects on AZO films deposited onto the mesoporous silicon substrate and shown a white light emission from the resulting composites after irradiating with high-energy nickel ions. Swift heavy ion (SHI)-induced morphological and structural changes, in terms of XRD and scanning electron microscopy, have also been studied. The emission after 325-nm excitation from a xenon arc lamp has been compared with the cathodoluminescence (CL) in studying the modifications in the deep-level defects induced by the high-energy radiations. Comparison between the low- and high-porosity mesoporous substrates is also presented. The nanocomposites are found to retain the crystalline structure after irradiation.

## Methods

PS samples were fabricated by wet electrochemical etching of p^*++*^-type Si(100) wafers with a resistivity of 0.01 to 0.05 Ω cm and at different current densities of 10 (LP) and 70 mA/cm^2^ (HP) using a 3:7:1 solution of HF/ethanol/glycerol. The porosity of samples LP (low porosity) and HP (high porosity) was 50 % and 70 %, respectively. The thickness of both samples was kept to 7 μm. After the fabrication, the samples were rinsed with ethanol and dried in pentane.

In order to study the effect of PS on PL and other structural and transport properties, AZO films were deposited by radio frequency magnetron sputtering. A sputtered target with a mixture (2 wt.% Al_2_O_3_-ZnO) was used, and the PS substrate temperature was kept at 300 °C during the deposition of the AZO film with a thickness of 150 nm. After deposition, the low- and high-porosity PS-AZO composites were named as ZLP and ZHP, respectively. As-deposited films were later annealed at 700 °C for 1 h in the tubular furnace in argon atmosphere. The annealed films were irradiated with 130-MeV nickel ions using the 15UD Pelletron Accelerator at the Inter University Accelerator Centre, New Delhi. The samples were mounted on a rectangular-shaped ladder and were irradiated in high vacuum chamber. The focused ion beam was scanned over an area of 1 × 1 cm^2^. The films of low porosity (ZLP) were irradiated with fluences of 1 × 10^12^ and 3 × 10^13^ ions/cm^2^, and the films of high porosity (ZHP) were irradiated with fluences of 3 × 10^12^ and 1 × 10^13^ ions/cm^2^. The beam current was kept constant at approximately 1.5 pnA. The electronic stopping power (energy dissipated in electronic excitations) and nuclear stopping power (energy dissipated in atomic collisions) by such ions in ZnO are around 24.63 and 0.44 keV/nm, respectively (calculated using SRIM2003 simulation code). The modifications in the properties of ZnO films are expected to be mainly due to the electronic excitations, though the contributions of small nuclear stopping power could not be ignored.

The structural properties and the thickness of the PS and nanocomposites were analyzed using a high-resolution field emission scanning electron microscope (SEM; JSM-7401 F, JEOL Ltd., Akishima-shi, Japan). The orientation and crystallinity of the ZnO crystallites were analyzed using an X-ray diffractometer (X'Pert PRO, PANalytical B.V., Almelo, The Netherlands) using CuKα radiation having a wavelength of 1.54 Å. PL properties were studied using a Varian Fluorescence spectrophotometer (Cary Eclipse, Agilent Technologies, Inc., Santa Clara, CA, USA) under the excitation by 325-nm photons using a 500-W xenon lamp. CL spectroscopy was done using JEOL JSM 5300 SEM with an electron beam energy of 15 keV. CL measurements were performed at 100 K in the UV-visible spectral range using a Hamamatsu R928P photomultiplier (Hamamatsu, Japan). A SPEX 340-E computer-controlled monochromator (Metuchen, NJ, USA) was used for the spectral analysis.

## Results and discussion

### X-ray diffraction

**Figure 1:**
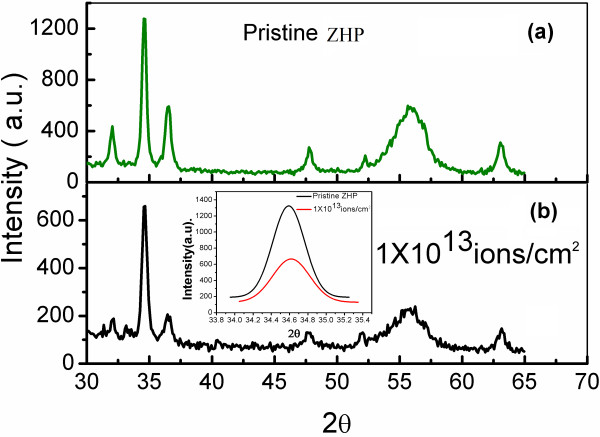
**XRD spectra of PS-AZO nanocomposite corresponding to ZHP (PS substrate fabricated at 70 mA/cm**^**2**^**).** (**a**) Pristine and (**b**) after irradiation at a fluence of 1 × 10^13^ ions/cm^2^. The inset shows the comparison of the (002) peak before and after irradiation.

The crystalline nature of the PS-AZO film was studied through XRD spectra (Figure 1) of pristine and irradiated samples at different fluences. The pristine PS-AZO film had a polycrystalline hexagonal wurtzite structure as confirmed by the dominant (002) peak. The ZHP sample, after irradiation at a fluence of 1 × 10^13^ ions/cm^2^, demonstrates a relative decrease (of about 50 %) in the peak intensities corresponding to the zinc oxide (i.e., (100), (002), and (101) peaks) and PS (at 2*θ* = 55°), indicating a decrease in the degree of crystallinity of the composite, after irradiation. Apart from this, a slight shift of 2*θ* = 0.02° in the (002) peak is observed after applying Gaussian fit and is attributed to the release of strain in the crystallites, similar to the effect reported earlier by Rehman et al. [18] (shown as inset in Figure 1b). Applying Scherrer's formula for the (002) peak, the crystallite size is found to decrease from 23.74 to 20.46 nm. The decrease in the crystallite size at a fluence of 1 × 10^13^ ions/cm^2^ could be attributed to the sputtering induced by the SHI irradiation, which is in accordance with the effect observed by some other groups [12,20,21]. The changes in the peak intensities for the samples with the low-porosity PS (10 mA/cm^2^) substrate show similar but less intense effects after irradiation at a fluence of 3 × 10^13^ ions/cm^2^. A relative dependence of the crystalline quality can be attributed to the substrate porosity dependence of ZnO grain growth [11] and its physical stability. The presence of the low-porosity silicon substrate tends to contribute in the retention of the physical and optical characteristics (shown in the later part of the manuscript). The above-mentioned result can be inferred as an opening for the possible optoelectronic application of PS-AZO films under harsh radiation conditions.

### Measurement of bandgap by optical method

For determining the bandgap of the ZnO film, the absorption coefficient (*α*) is obtained from transmittance data using the following equation:

$\alpha =-\frac{1}{d}\text{ln }T\text{,}$ where *d* is the thickness of the film and *T* is the optical transmittance.

**Figure 2:**
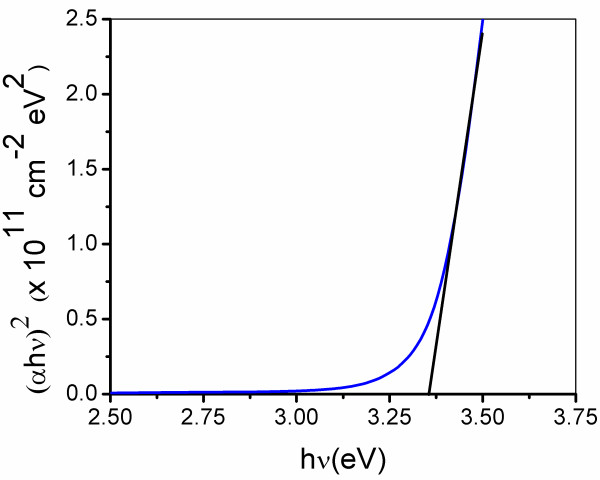
**Bandgap calculation of AZO film from UV–vis spectroscopy.** (αhν)^2^ vs. hν (photon energy) plot of zinc oxide film deposited over the glass substrate.

The bandgap can be estimated using the equation

(1) $${\left(\text{αhν}\right)}^{2}=A(\text{hν}-E_{\text{g}})\text{,}$$

where *h* is the Planck constant, *ν* is the frequency of the incident photon, and *A* is a constant. Hence, from Figure 2, the estimated optical bandgap of the film is 3.35 eV, involving direct electronic transitions [22,23].

### Scanning electron microscopy

**Figure 3:**
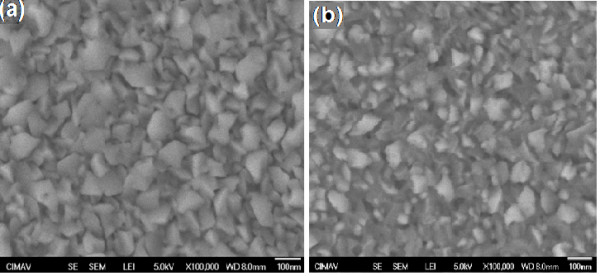
**SEM images corresponding to ZHP (PS-AZO).** (**a**) Pristine and (**b**) after irradiation at a fluence of 1 × 10^13^ ions/cm^2^.

In order to check the morphological changes, secondary electron microscope images (Figure 3) were analyzed before (pristine) and after irradiation for the ZHP sample. Before irradiation, the films are found to have regular flaky grains spread uniformly over PS. The size of the grains before irradiation appears to be uniform and is in the range of 50 to 90 nm. However, the irradiated film (at a fluence of 1 × 10^13^ ions/cm^2^) shows apparent changes in the morphology which could be attributed to the inelastic collisions between the electrons of the target material and the high-energy ions. Most of the flaky structure appears as irradiation-induced dark patches (Figure 3b). The observed phenomenon after irradiation is similar to the one reported by Rehman et al. [20]. The observed changes in the morphology could be co-related to the results of XRD studies.

### Photoluminescence spectroscopy

**Figure 4:**
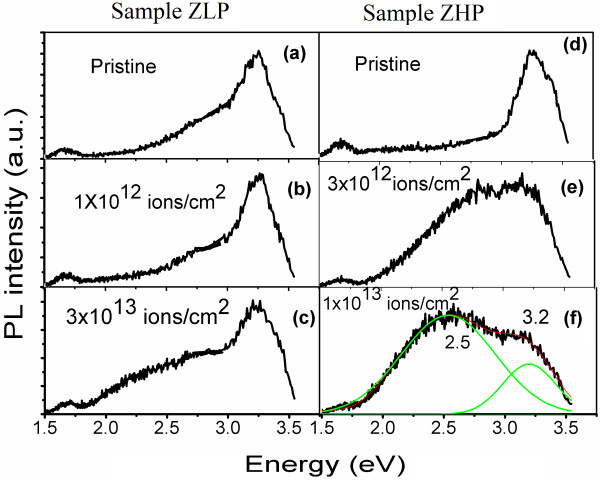
**PL spectra of PS-AZO nanocomposites irradiated at different fluences.** (**a**) Pristine, (**b**) irradiated with 1 × 10^12^ ions/cm^2^, and (**c**) 3 × 10^13^ ions/cm^2^ correspond to the ZLP sample. (**d**) Pristine, (**e**) irradiated with 3 × 10^12^ ions/cm^2^ and (**f**) 1 × 10^13^ ions/cm^2^ correspond to the ZHP sample.

The photoluminescence (PL) characteristics of the PS-AZO nanocomposite were studied (Figure 4) before and after SHI irradiation using nickel ions with a low-energy beam of 130 MeV. Figure 4a shows the PL spectrum of the composite formed with the ZLP substrate, with a peak around 3.25 eV attributed to the band-edge excitonic transition of ZnO [24,25]. The PL emission peak shows a low-intensity shoulder/tapering end at the left-hand side of the peak which can be attributed to the well-known oxygen vacancies in the zinc oxide layer [26-28]. Although the origin of this broad defect emission is controversial, it has been attributed to the singly ionized oxygen vacancies (Vo^−^) [27] and interstitial oxygen (O_i_) [27,28]. Particularly, a ZnO emission of 2.5 eV has been assigned to the recombination of delocalized electrons close to the conduction band with deeply trapped holes in Vo^+^ centers. Figure 4b shows PL emission from the PS-AZO nanocomposite with low-porosity substrate (i.e., made with a current density of 10 mA/cm^2^) after irradiation at a fluence of 1 × 10^12^ ions/cm^2^. No significant change in the defect-related PL intensity around 2.5 eV was observed, proving the stability of the structure up to a certain degree of irradiation fluences. On the other hand, an increase in the degree of fluence to 3 × 10^13^ ions/cm^2^ generated a significant increase of the PL intensity corresponding to the defects centered at 2.5 eV in Figure 4c, which is attributed to an increase in the deep-level defects, possibly oxygen vacancies. The formation of defects can be further understood as follows: As the SHI penetrates the solid, inelastic collisions are expected between the ion and the target electrons. In general inelastic collision, it is believed that electronic excitation and ionization of the target atoms plays a dominant role for high-energy heavy ion impact on ZnO. In the case of SHIs, inelastic nuclear collision results in the energy transfer which can yield localized heating/amorphization and a significant increase in the number of defects. On the other hand, due to the fact that in this energy range, electronic stopping is much bigger than nuclear stopping, electronic excitation caused by strong electronic stopping can weaken oxygen bonds, resulting in the formation of oxygen vacancies [29].

Although a similar behavior was observed for the ZHP sample as well (see Figure 4e,f), an increase of fluence is shown to enhance significantly the defect-related emission centered at 2.5 eV. The peaks corresponding to 2.5 to 3.25 eV have been observed to merge, resulting in the formation of broad white emission band from 3.25 to 1.50 eV. In comparing Figure 4e,f, a further increase in the level of fluence is found to increase the signal corresponding to the defects (2.5 eV) with a simultaneous decrease in the band-edge emission (3.25 eV). The intensity of the emission band corresponding to PS (around 1.7 eV) is found to decrease slightly with an increase in the level of fluence which is in accordance to the already reported work of Singh et al. [19]. The resulting PL spectra are observed to have an almost white emission from the composite structure which can be very useful for the display devices.

### Cathodoluminescence

**Figure 5:**
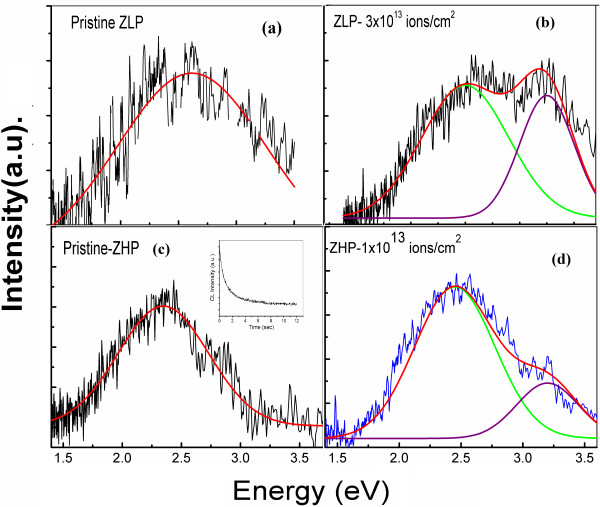
**CL spectra of PS-AZO nanocomposites irradiated at different fluences.** (**a**) Pristine ZLP sample. (**b**) ZLP sample irradiated with 3 × 10^13^ ions/cm^2^. (**c**) Pristine ZHP sample. (**d**) ZHP sample irradiated with 1 × 10^13^ ions/cm^2^.

In order to further investigate the defect-related luminescence mechanisms, CL spectroscopy was performed on all the composite samples. The main advantage of the CL is the spacial resolution, determined by the distribution of the excess carrier in the material and is therefore not limited by the diffraction in the collection and excitation optics, which is very customary in all the far-field techniques. Hence, to enhance our understanding regarding the optical properties of PS-ZnO composites under irradiation conditions, the PL studies have been complimented by CL analysis. The major reason that very few studies reported CL of PS is due to an extremely weak and unstable data. The inset in Figure 5c shows a strong decrease of the CL emission signal of the PS produced by the electron beam irradiation in SEM. Deposition of AZO layer on the mesoporous silicon layer leads to the stability of the composite structure. As the electron beam can inject into the samples, a strong charge density produces saturation of radiative levels; hence, the luminescence from defects can be explored in detail. Figure 5a shows CL spectra from the non-irradiated (pristine) sample with a broad emission centered at about 2.6 eV, which apparently corresponds to the ZnO defect emission centered at 2.5 eV in PL spectra. The low-intensity CL recorded in the spectra is attributed to the low sensibility of our system at room temperature. As compared to the PL spectra from the pristine sample, the ZnO band edge emission was not resolved in CL spectra, possibly due to presence of a high density of defects in the film-substrate interface. Figure 5b shows the CL spectra from the irradiated ZLP sample, revealing two emissions centered at 3.2 and 2.5 eV, associated to the ZnO band edge and defect emission. As commented in the ‘Photoluminescence spectroscopy’ section, the 2.5 eV band is associated to the presence of oxygen vacancies and other native defects [30-32]. The CL spectrum of the ZHP sample shows a similar behavior as that of the ZLP sample (Figure 5c,d), with the generation of the ZnO band edge (3.2 eV) after the SHI irradiation. This effect is not correlated with PL measurements, where the relative intensity of UV emission decreased after irradiation (Figure 4c,f). In CL measurements, an increase of the relative intensity of the ZnO band edge emission typically corresponds to an improvement of the crystalline quality, mainly due to annealing effects [30,33]. Possibly, the SHI irradiation generated a high density of point defects mostly at the film's surface, which was recorded in PL spectra with a relative increase of the 2.5 eV emission (Figure 4c,f), while at the film-substrate interface, the irradiation-generated heating produces annealing of defects (enhancement of crystalline behavior) as shown in the corresponding CL spectra (Figure 5b,d).

### Electrical properties

**Figure 6:**
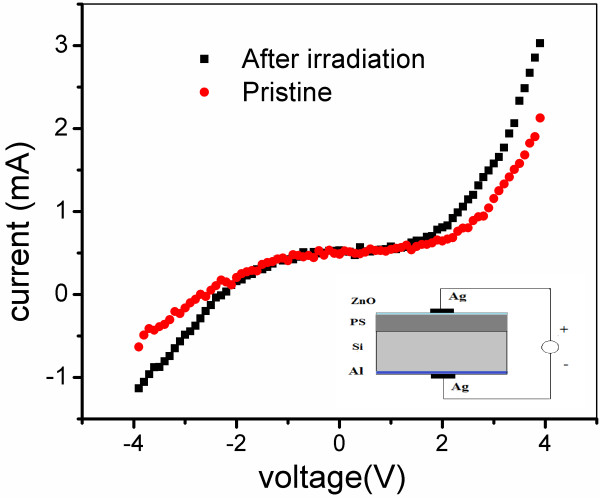
**I-V curves of ZnO-PS structure (ZHP sample) in a sandwich configuration, measured at room temperature.** The voltage varies from −4 to 4 V with a sweep rate of 150 mV/s. The inset in the lower part shows the schematic of the configuration used for I-V characteristics. The inset of Figure 5c shows CL emission spectra as a function of time showing the degradation of the as-etched PS layer by high-energy electrons.

Figure 6 demonstrates the non-ohmic electrical response of the current–voltage (I-V) measurements performed in a two-terminal AZO-PS-Si configuration (inset of Figure 6) which is normally attributed to the formation of Schottky barriers at the ZnO-PS interfaces. It was seen that after irradiation at 1 × 10^13^ ions/cm^2^, the ZHP sample shows little higher forward/reverse current than the pristine sample. In order to quantify the deviation from the Schottky behavior, the rectifying factor (*I*_F_/*I*_R_) was calculated at 4 V and was found to be 3.34 and 2.62 for the pristine ZHP sample and that after irradiation (1 × 10^13^ ions/cm^2^ of fluence), respectively. A slight reduction in the rectifying factor after irradiation can be attributed to the irradiation-induced thermal annealing of the defects and hence an improvement of the crystallinity at the interface of ZnO-PS.

## Conclusions

We report the substrate porosity and fluence-dependent white light emission from RF-sputtered zinc aluminum oxide deposited on PS after SHI irradiation. The structures are shown to partially retain their crystallinity, when irradiated with light Ni ions. Composites are found to have a rectifying behavior which reduces by a factor of 0.78 after irradiation. Its stability under harsh irradiation conditions makes it useful for space applications. Apart from that, the tunability in the optical properties is important for optoelectronic applications.

## Competing interests

The authors declare that they have no competing interests.

## Authors' contributions

YK made the porous silicon samples and performed their characterization before and after irradiation. MHZ participated in performing the cathodoluminescence studies and their corresponding analysis. SFOM participated in acquiring the SEM and XRD facilities. FS performed the SHI irradiation at IUAC, New Delhi. XM participated in the deposition of AZO film on PS and glass substrates. VA conceived the study, participated in its design and coordination, and codrafted the manuscript with YK. All authors read and approved the final manuscript.

## Authors' information

YK is a Ph.D. student registered at CIMAV, Chihuahua, and is doing his research work at CIICAp, UAEM, Mexico. MHZ is an associate professor at CNyN, UNAM and is working on the characterization of semiconductor nanostructures. SFOM is working as a researcher at CIMAV, Chihuahua, in the field of nanostructured materials. FS is a scientist at IUAC, New Delhi. XM is a senior scientist at CIE, UNAM working on thin film semiconductors for photovoltaic applications.VA is working as a professor investigator at CIICAp, UAEM in the field of nanostructured silicon.

## References

[B1] Pan ZW Dai ZR Wang ZL Nanobelts of semiconducting oxides Science 2001 291 1947 1949 10.1126/science.1058120 11239151

[B2] Lin CH Chiou BS Chang CH Lin JD Preparation and cathodoluminescence of ZnO phosphor Mat Chem Phys 2003 77 647 654 10.1016/S0254-0584(02)00120-7

[B3] Kashyout A Soliman M El Gamal K Fathy M Preparation and characterization of nano particles ZnO films for dye-sensitized solar cells Mater Chem Phys 2005 90 230 233 10.1016/j.matchemphys.2004.11.031

[B4] Schuber EF Kim JK Solid-state light sources getting smart Science 2001 308 1274 1278 10.1126/science.1108712 15919985

[B5] Nanto H Minami T Shooji S Takata S Electrical and optical properties of zinc oxide thin films prepared by rf magnetron sputtering for transparent electrode applications J Appl Phys 1984 55 1029 1034 10.1063/1.333196

[B6] Tang W Cameron DC Aluminum-doped zinc oxide transparent conductors deposited by the sol–gel process Thin Solid Films 1994 238 83 87 10.1016/0040-6090(94)90653-X

[B7] Kim KH Park KC Ma DY Structural, electrical and optical properties of aluminum doped zinc oxide films prepared by radio frequency magnetron sputtering J Appl Phys 1997 81 7764 7772 10.1063/1.365556

[B8] Bsiesy A Cox TI Canham L Electroluminescence from porous silicon using liquid contacts Properties of Porous Silicon 1997 INSPEC Publication, London 283 289

[B9] Canham LT Canham L Visible photoluminescence from porous silicon Properties of Porous Silicon 1997 INSPEC Publication, London 249 255

[B10] Wang C-F Ho B Yi H-H Li W-B Structure and photoluminescence properties of ZnS films grown on porous Si substrates Opt Laser Technol 2011 43 1453 1457 10.1016/j.optlastec.2011.04.018

[B11] Yogesh K Escorcia Garcia J Fouran S Olive-Méndez SF Sivakumar VV Kanjilal D Agarwal V Influence of mesoporous substrate morphology on the structural, optical and electrical properties of RF sputtered ZnO layer deposited over porous silicon nanostructure Appl Surf Sci 2010 258 2283 2288

[B12] Zang H Wang ZG Peng XP Song Y Liu CB Wei KF Zhang CH Yao CF Ma YZ Zhou LH Sheng YB Gou J Modification of ZnO films under high energy Xe-ion irradiations Nucl Instrum Methods Phys Res, Sect B 2008 266 2863 2867 10.1016/j.nimb.2008.03.131

[B13] Fouran S Kulriya PK Pivin JC Origin of swift heavy ion induced stress in textured ZnO thin films: an in situ X-ray diffraction study Solid State Commun 2010 150 1751 1754 10.1016/j.ssc.2010.07.026

[B14] Krasheninnikov AV Banhart F Engineering of nanostructured carbon materials with electron or ion beams Nat Mater 2007 6 723 733 10.1038/nmat1996 17906658

[B15] Krasheninnikov AV Nordlund K Ion and electron irradiation-induced effects in nanostructured materials J Appl Phys 2010 107 071301 071370 10.1063/1.3318261

[B16] Matsunami N Fukushima J Sakta M Okayasu S Sugai H Kakiuchida H Electrical property modifications of In-doped ZnO films by ion irradiation Nucl Instrum Methods Phys Res, Sect B 2010 268 3071 3075 10.1016/j.nimb.2010.05.045

[B17] Sugai H Matsunami N Fukuoka O Sataka M Kato T Okayasu S Shimura T Tazawa M Electrical conductivity increase of Al-doped ZnO films induced by high-energy-heavy ions Nucl Instrum Methods Phys Res, Sect B 2006 250 291 294 10.1016/j.nimb.2006.04.126

[B18] Singh RC Singh MP Singh O Chandi PS Kumar R Effect of 100 MeV O7+ ions irradiation on ethanol sensing response of nanostructures of ZnO and SnO2 Appl Phys A 2010 98 161 166 10.1007/s00339-009-5442-5

[B19] Singh RG Fouran Singh I Sulania D Kanjilal K Sehrawat V Agarwal RM Mehra: electronic excitations induced modifications of structural and optical properties of ZnO–porous silicon nanocomposites Nucl Instrum Meth Phys Res, Sect B 2009 267 2399 2402 10.1016/j.nimb.2009.04.005

[B20] Rehman S Singh RG Pivin JC Bari W Singh F Structural and spectroscopic modifications of nanocrystalline zinc oxide films induced by swift heavy ions Vaccum 2011 86 87 90 10.1016/j.vacuum.2011.04.019

[B21] Agarwal DC Avasthi DK Singh F Kabiraj D Kulariya PK Sulania I Pivin JC Chauhan RS Swift heavy ion induced structural modification of atom beam sputtered ZnO thin film Surf CoatTechnol 2009 203 2427 2431 10.1016/j.surfcoat.2009.02.109

[B22] Pankove JI Optical Processes in Semiconductors 1971 Dover, New York

[B23] Smith RA Semiconductors 1978 Cambridge University Press, Cambridge

[B24] Pal U Melendrez R Chernov V Flores MB Thermoluminescence properties of ZnO and ZnO:Yb nanophosphors Appl Phys Lett 2006 89 183118 183120 10.1063/1.2374866

[B25] Key A Gratzel M Low cost photovoltaic modules based on dye sensitized nanocrystalline titanium dioxide and carbon powder Sol Ener Mater Sol Cells 1996 44 99 117 10.1016/0927-0248(96)00063-3

[B26] Vanheusden K Seager CH Warren WL Tallant DR Voigt JA Correlation between photoluminescence and oxygen vacancies in ZnO phosphors Appl Phys Lett 1996 68 403 405 10.1063/1.116699

[B27] Lin B Fu Z Jia Y Green luminescent center in undoped zinc oxide films deposited on silicon substrates Appl Phys Lett 2001 79 943 945 10.1063/1.1394173

[B28] Xu PS Sun YM Shi CS Xu FQ Pan HB The electronic structure and spectral properties of ZnO and its defects Nucl Instrum Methods Phys Res, Sect B 2003 199 286 290

[B29] Hemon S Berthelot A Durfour C Domenges B Paumier E Irradiation of a tin oxide nanometric powder with swift heavy ions Nucl Instrum Methods Phys Res, Sect B 2000 166-167 927 932

[B30] González A Herrera M Valenzuela J Escobedo A Pal U CL study of yellow emission in ZnO nanorods annealed in Ar and O2 atmospheres Superlatt and Microst 2009 45 421 428 10.1016/j.spmi.2008.10.036

[B31] Dong YF Tuomisto F Svensson BG Kuznetsov AY Brillson L Vacancy defect and defect cluster energetics in ion-implanted ZnO Phys Rev B 2010 81 81201 81204

[B32] Mosbacker HL Zgrabik C Hetzer MJ Swain A Look DC Cantwell G Zhang J Song JJ Brillson LJ Thermally driven defect formation and blocking layers at metal-ZnO interfaces Appl Phys Lett 2007 91 072102 072103 10.1063/1.2772664

[B33] González A Herrera M Valenzuela J Escobedo A Pal U Cathodoluminescence evaluation of defect structure in hydrothermally grown ZnO:Sb nanorods J Nanoscience and Nanotech 2011 11 5526 5531 10.1166/jnn.2011.3424 21770214

